# Cyanidin-3-O-Glucoside Ameliorates Palmitic-Acid-Induced Pancreatic Beta Cell Dysfunction by Modulating CHOP-Mediated Endoplasmic Reticulum Stress Pathways

**DOI:** 10.3390/nu14091835

**Published:** 2022-04-28

**Authors:** Yunan Chen, Xueyan Li, Lei Su, Qianrong Hu, Wenli Li, Jialin He, Lina Zhao

**Affiliations:** 1 Department of Nutrition, School of Public Health, Sun Yat-sen University (Northern Campus), Guangzhou 510080, China; chenyn256@mail2.sysu.edu.cn (Y.C.); lixy269@mail2.sysu.edu.cn (X.L.); sulei5@mail2.sysu.edu.cn (L.S.); huqianr@mail2.sysu.edu.cn (Q.H.); liwli7@mail2.sysu.edu.cn (W.L.); hejlin8@mail2.sysu.edu.cn (J.H.); 2 Guangdong Provincial Key Laboratory of Food, Nutrition and Health, Guangzhou 510080, China

**Keywords:** Cyanidin-3-O-glucoside, palmitic acid, C/EBP homologous protein, endoplasmic reticulum stress, beta cell function, type 2 diabetes mellitus

## Abstract

Cyanidin-3-O-glucoside (C3G) is a natural colorant with anti-diabetic properties, while its underlying mechanisms remain far from clear. Here, we investigated the protective role of C3G on palmitic acid (PA)-induced pancreatic beta cell dysfunction and further decipher its possible molecular mechanisms. Both primary isolated mouse islets and the INS-1E cell were used, and treated with a mixture of PA (0.5 mM) and C3G (12.5 µM, 25 µM, 50 µM) for different durations (12, 24, 48 h). We found that C3G could dose-dependently ameliorate beta cell secretory function and further alleviate cell apoptosis. Mechanistically, the primary role of the PKR-like ER kinase (PERK) endoplasmic reticulum (ER) stress pathway was detected by RNA sequencing, and the PERK-pathway-related protein expression, especially the pro-apoptotic marker C/EBP homologous protein (CHOP) expression, was significantly downregulated by C3G treatment. The critical role of CHOP in mediating the protective effect of C3G was further validated by small interfering RNA. Conclusively, C3G could ameliorate PA-induced pancreatic beta cell dysfunction targeting the CHOP-related ER stress pathway, which might be used as a nutritional intervention for the preservation of beta cell dysfunction in type 2 diabetes mellitus.

## 1. Introduction

The widespread epidemic of type 2 diabetes mellitus (T2DM), affecting over 10.5% of the global population, causes a huge burden for public health [1]. T2DM features both insulin resistance and pancreatic beta cell dysfunction. The progression from the insulin resistance state to overt diabetes or hyperglycemia state is determined by the severity of pancreatic beta cell dysfunction, which fails to compensate for systemic insulin resistance [2,3]. Elevated free fatty acids (FFA, palmitic acid as a typical example) accompanied by insulin resistance states, are known to exert a chronic damaging effect on pancreatic beta cells (briefly called lipotoxicity), even before the glucotoxicity and are recognized as an independent diabetogenic factor [4]. Preservation or protection of beta cell function from deleterious factors is crucial to the early prevention and treatment of diabetes [5]. A growing body of literature has suggested that plant-derived polyphenols have great potential for preserving the physiological functions of beta cells and restoring the negative effects of lipotoxicity, which might be able to be used as a precision nutritional intervention for diabetes [6].

Cyanidin-3-O-glucoside (C3G), the most abundant anthocyanin, derived from deep-colored fruits and vegetables, belongs to the polyphenol of the flavonoid group [7,8]. The multiple health-prompting effects of anthocyanins have attracted great focus in the past two decades [9,10]. Accumulating epidemiological evidence from both large prospective cohort studies and intervention studies strongly supported that higher dietary anthocyanin consumption was associated with a lower risk of diabetes [11,12], while the underlying molecular mechanism of the anti-diabetic effect of C3G was still far from clear. Most of the published experimental studies on the anti-diabetic effect of C3G focused on its protective effect on peripheral insulin resistance. It has been reported that C3G exerts a protective effect on the liver, muscle, and adipose tissue through a variety of mechanisms, including regulating adipokine secretion, reducing the inflammatory response, activating AMPK signaling, among others [9]. Only several published studies have focused on the effect of C3G on the beta cell, which is also one of the target cells of C3G [13]. C3G has been reported to be able to protect/improve the viability of pancreatic beta cells or islets from various deleterious factors, including H_2_O_2_ exposure, glucotoxicity, and amylin or Ab1-42 treatment [14,15,16,17], while the protective role of C3G on palmitic-acid-induced beta cell dysfunction and its possible mechanisms have not been reported yet. In the present study, we systemically explored the dose-dependent effect of C3G on palmitic-acid-caused beta cell dysfunction, in both primary isolated islets and beta cell lines. To fully decipher the molecular responses underneath the protective effect of C3G on palmitic-acid-induced pancreatic beta cell dysfunction, we applied RNA sequencing (RNA-seq) to identify all differentially expressed genes after treatment of C3G, and further verified the critical mechanisms using Western blot (WB) and small interfering RNA (siRNA).

## 2. Materials and Methods

### 2.1. Materials

Cyanidin-3-O-glucoside was purchased from TAUTO BIOTECH (Shanghai, China). Palmitic acid (PA) and fatty acid-free-BSA were purchased from Sigma-Aldrich (St. Louis, MI, USA).

### 2.2. Cell Culture and Glucose-Stimulated Insulin Secretion Assay

The rat insulinoma INS-1E cell line was provided by FENGHUISHENGWU, Ltd. (Hunan, China). INS-1E cells were maintained in RPMI 1640 supplemented with 10% fetal bovine serum (FBS), 1% penicillin/streptomycin (Gibco, Carlsbad, CA, USA), 1% 1 mM sodium pyruvate, 10 mM HEPES (Solarbio Life Sciences, Beijing, China), and 0.05 mM 2-mercaptoethanol (Sigma-Aldrich, St. Louis, MI, USA).

Islets of Langerhans were isolated from C57BL/J6 male mice aged 8–12 weeks as previously described and were approved by the Animal Care and Utilization Committee of Sun Yat-sen University (Ethics Committee Approval No. 2021-010) [18]. Briefly, the pancreases of mice were fully expanded with 0.5 mg/mL collagenase P (Roche, Shanghai, China) dissolved in Hanks’ balanced salt solution (Gibco, Carlsbad, CA, USA) and cultured in RPMI 1640 containing 10% FBS and 1% penicillin/streptomycin. After 4 h at 5% CO_2_ and 37 °C, islets with intact borders were collected to use for glucose-stimulated insulin secretion assay (GSIS) experiments. Cell viability was measured by the CCK8 assay (Dojindo, Kumamoto, Japan) and the process was performed according to the manufacturer’s instructions.

For GSIS assay, INS-1E cells were grown in 12-well plates for two days and primary islets were collected in individual tubes for instant use (10 islets/tube), after which cells/islets were treated with 0.5 mM PA prepared as previously described [19] with or without various concentrations of anthocyanin (12.5 µM, 25 µM, 50 µM) for different time points (12 h, 24 h, or 48 h). The control group was treated with fatty-acid-free BSA. At the end of the treatment, the cell culture medium was replaced with PH 7.4 glucose-free KRBH (Krebs–Ringer bicarbonate HEPES buffer, containing 10 mM HEPES, 129 mM NaCl, 4.8 mM KCl, 1.2 mM MgSO_4_·7H_2_O, 1.2 mM KH_2_PO_4_, 2.5 mM CaCl_2_, 5 mM NaHCO_3_, and 0.1% Fatty-acid-free BSA) and incubated for an hour at 37 °C. Then, the glucose-free KRBH was discarded and incubated with 3 mM and 25 mM glucose KRBH for one hour each in turn, and the supernatants were collected for insulin assays (ELISA kit assay, Mercodia, Uppsala, Sweden). The glucose concentrations were based on previous studies on pancreatic beta cell function [20,21]. Cells were lysed in RIPA lysis to collect protein in normalization.

### 2.3. Apoptosis Assessment by Flow Cytometry and Immunofluorescent Staining

Cell apoptosis was examined by flow cytometry using the Annexin V-FITC/propidium iodide (PI) apoptosis detection kit (Elabscience, Wuhan, China). INS-1E cells were seeded in 6-well plates treated with BSA, PA, or PA plus 50 µM C3G for 24 h. Then, cells were suspended with Annexin V Binding Buffer (500 µL/5 × 10^5^ cells) with 5 µL Annexin V-FITC and 5 µL PI added at the end of the intervention. After being gently vortexed, the mixture needed an incubation for 20 min at room temperature avoiding light. Finally, the cell apoptosis rate was determined by utilizing an EPICS XL-MCL flow cytometer (BECHMAN COULTER, Brea, CA, USA) and analyzed by FlowJo™ software (BD Biosciences, New York, NY, USA). Annexin V binds to the membrane of early apoptotic cells and PI stains the DNA of late apoptotic or necrotic cells; thus, the apoptotic cells include early apoptotic cells (Annexin V+/PI−) and late apoptotic cells (Annexin V−/PI+).

Cell apoptosis rate was further confirmed by 4′-6-diamidino-2-phenylindol (DAPI)/PI immunofluorescent staining (Beyotime, Shanghai, China). INS-1E cells were grown on glass coverslips on 35 mm confocal dishes and after 36 h the cells were treated with BSA only, 0.5 mM PA, or PA containing 50 µM C3G. One hour prior to the end of treatment, 10 µg/mL of PI and 20 µg/mL of DAPI were added to the culture medium. At the end of incubation, cells were washed 3 times with clean PBS and fixed with 4% paraformaldehyde at 4 °C for 15 min. Cells were mounted with antifade mounting medium (Beyotime, Shanghai, China) and observed under a laser scanning confocal microscope (Leica TCS SP5, Wetzlar, Germany). Random images were acquired from different sites within the confocal dish and image analysis was performed to calculate cell apoptosis rate based on the ratio of the fluorescence area of PI (staining late apoptotic and necrotic cells) to DAPI (staining nuclei of all cells).

### 2.4. RNA Sequencing and Quantitative Real-Time PCR

INS-1E cells were seeded in 6-well plates treated with BSA, PA, or PA plus 50 µM C3G for 24 h. At the end of treatment, total RNA was extracted utilizing Trizol reagent (Thermo Fisher, Waltham, MA, USA) with three replicate samples collected for each group and sent to LC Bio (Zhejiang, China) for further processing of the samples and RNA sequencing (RNA-seq) analysis. Briefly, total RNA was quantified and the mRNA within was purified using Dynabeads Oligo (dT) (Thermo Fisher, Waltham, MA, USA). Then, mRNA was fragmented and reverse-transcribed to cDNA by SuperScript™ II Reverse Transcriptase (Invitrogen, cat.1896649, Carlsbad, CA, USA). Finally, the processed products were amplified with PCR, and the 2 × 150 bp paired-end sequencing (PE150) was performed on an Illumina Novaseq™ 6000 (LC-Bio Technology Co., Ltd., Hangzhou, China) following the vendor’s recommended protocol. The differentially expressed genes (DEGs) were selected with fold change >2 and statistically significant (*p* < 0.05) genes to make heatmap, volcano maps, and Gene Set Enrichment Analysis (GSEA) enrichment plots using the OmicStudio tools (https://www.omicstudio.cn/tool, the accessed date was 9 October 2021). The assigned GEO accession number is GSE196704.

Quantitative real-time PCR (qPCR) was performed as previously described [22]. Briefly, the concentration and purity of total RNA were measured. cDNA was acquired using a PrimeScript™ RT reagent Kit with gDNA Eraser (Takara, Kyoto, Japan), and TB Green^®^ Premix Ex Taq™ II plus ROX Reference Dye II (Takara, Kyoto, Japan) was applied to perform qPCR on an Applied Biosystems Prism 7000 sequence detection system. The target primers were designed based on mRNA sequences given in NCBI Genebank and were verified on NCBI-primer blast.

The primers were: toll-like receptor 4 (TLR4) Sense: GATCTGAGCTTCAACCC, Anti-sense: TTGTCTCAATTTCACACCTGGA; G-protein-coupled receptor 40 (GPR40) Sense: GCTGCCTTCCCCTTTGGATA, Anti-sense: TGTTGATGCCCAGGGAACTG; C/EBP homologous protein (CHOP) Sense: CCCCAGGAAACGAAGAGGAAG, Anti-sense: AATCTGGAGAGCGAGGGCTT; glucose transporter 2 (GLUT2) Sense: AATTTGGACCGGCACATGCT, Anti-sense: CTGAGGCCAGCAATCTGACTA; PKR-like ER kinase (PERK) Sense: GCGGCAGTGAGAAGTGGAAT, Anti-sense: ACCTTCCAATCAGCAACGGA; β-actin Sense: ATATCGCTGCGCTCGTCGT, Anti-sense: CATACCCACCATCACACCCTGG.

### 2.5. Western Blot

Whole-cell protein was extracted by RIPA Lysis Buffer with Halt™ Protease and Phosphatase Inhibitor Single-Use Cocktail, EDTA-Free (100×), and protein concentration was measured by Pierce BCA Protein Assay Kit (All from Thermo Scientific™, Waltham, MA, USA). Total protein was separated by SDS-PAGE, transferred to PVDF membrane (Millipore, Billerica, MA, USA), and detected by SuperSignal™ West Femto Maximum Sensitivity Substrate (Thermo Scientific™, Waltham, MA, USA). Primary and secondary antibodies used in the experiments were listed as follows: anti-PERK, anti-Phospho-PERK (Thr980), anti-glucose-regulated Protein 78 (GRP78), anti-CHOP, anti- eukaryotic initiation factor 2 alpha (eif2α), anti-elF2 alpha (phospho S52), anti-cleaved-caspase-3, anti- B cell lymphoma 2 (BCL2)-associated X protein (BAX), anti-BCL2 (1:1000 dilution, Cell Signaling Technology, Danfoss, MA, USA), mouse anti-rabbit IgG-HRP, and m-IgGκ BP-HRP (1:3000 dilution, Santa Cruz Biotechnology, Santa Cruz, CA, USA).

### 2.6. Inflammatory Factors Assay

The supernatant of INS-1E cells was collected after the intervention mentioned in 2.3 to detect inflammatory factor (interleukin-1beta, tumor necrosis factor-alpha) levels using the ELISA kit (NEOBIOSCIENSE, Shenzhen, China). The detailed procedures were followed in accordance with the manufacturer’s instructions.

### 2.7. Small Interfering RNA Transfections

CHOP small interfering RNA (siRNA) oligos with control oligos, and RNAFit transfection reagent were provided by HANBIO (Wuhan, China). The transfection process was performed according to the manufacturer’s instructions. After 36 h of transfection, INS-1E cells were then exposed to PA or C3G for GSIS assay and were collected for WB as described above.

### 2.8. Statistical Analysis

Data were shown as mean ± SEM, and one-way ANOVA followed by LSD post hoc test was utilized for comparing differences between multiple groups. All experiments were performed at least in biological triplicate. SPSS 25.0 (Chicago, IL, USA) was used for all analyses. For WB and immunofluorescence data, representative images were shown in all figures. *p* < 0.05 was taken to be statistically significant.

## 3. Results

### 3.1. C3G Directly Ameliorated PA-Induced Pancreatic Beta Cell Dysfunction in Vitro

**Figure 1:**
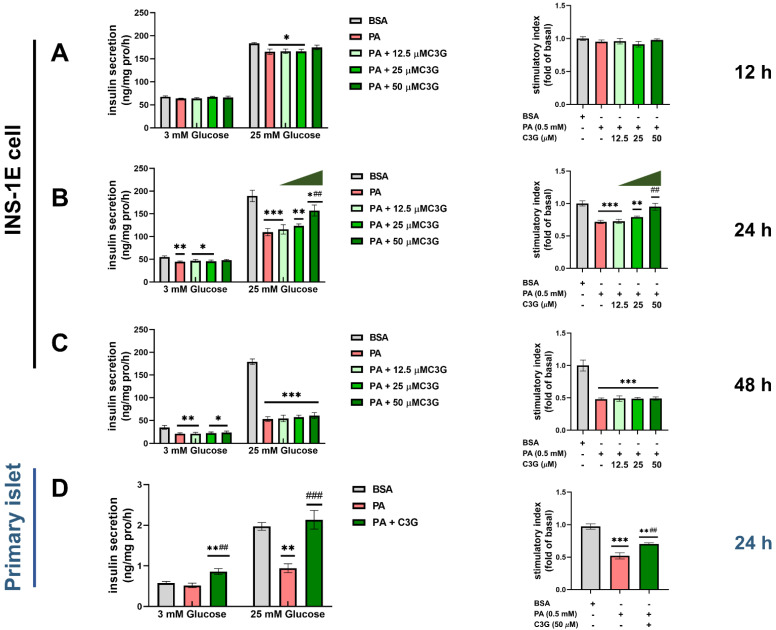
Effects of C3G on glucose-stimulated insulin secretion (GSIS) in INS-1E cells and mouse primary islets under palmitic acid (PA) exposure. (**A**–**C**) GSIS in INS-1E cells after treated with 0.5 mM PA and with or without different doses of C3G (12.5 µM, 25 µM, 50 µM) for different time points (12 h, 24 h, and 48 h). The stimulatory index was expressed as fold change versus the BSA group (the control group treated with BSA). (**D**) GSIS of mouse primary islets with 0.5 mM PA plus 50 µM C3G for 24 h treatment. Experiments were performed in triplicate and results were shown as means ± SEM, * *p* < 0.05, ** *p* < 0.01, *** *p* < 0.001 versus BSA control group, ## *p* < 0.01, ### *p* < 0.001 versus PA group.

To determine whether C3G could protect the secretory function of the pancreatic beta cell, we first treated INS-1E cells with 0.5 mM PA in combination with a gradient concentration of C3G (12.5 µM, 25 µM, 50 µM) for different time points (12 h, 24 h, 48 h) (Figure 1A–C). No cytotoxicity was observed at selected dosages (Figure S1A). A dose-dependent protective effect of C3G on PA-induced beta cell secretory dysfunction was observed at 50 µM C3G treatment for 24 h. Meanwhile, GSIS (25 mM glucose) in the PA + C3G group rose by 43% and the stimulatory index augmented by 33% compared to the PA group, respectively (Figure 1B). Next, 50 µM C3G treated for 24 h was used in the following experiments.

Then, we further confirmed the above observation in the primary isolated islets. Compared to the BSA group, GSIS was decreased by 52% due to PA exposure and that was significantly restored by C3G treatment, with GSIS increasing by 127% and stimulatory index increasing by 35%, respectively (Figure 1D).

**Figure 2:**
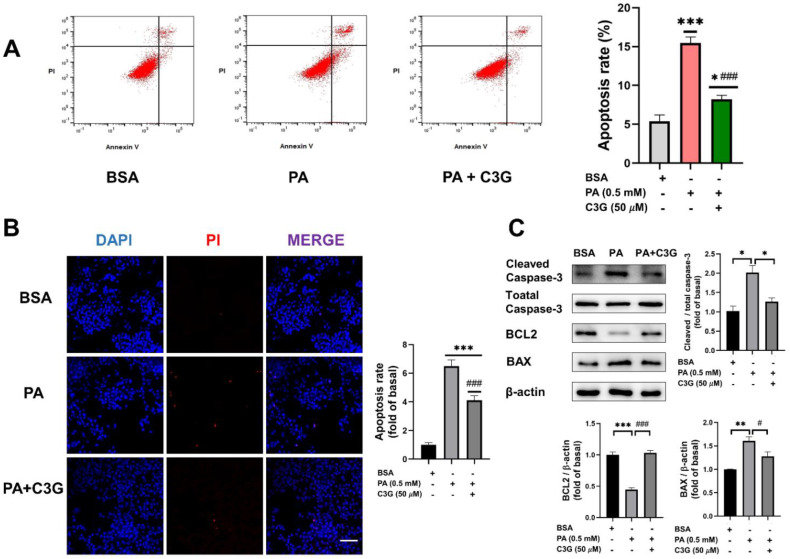
Effects of C3G on PA-induced apoptosis in INS-1E cells. (**A**) The apoptosis rate of INS -1E cells was assessed by Annexin V-FITC/propidium iodide (PI) double staining flow cytometry. (**B**) Apoptotic cells were detected by DAPI/PI fluorescent staining (40× oil, scale bar: 25 µM). Cell apoptosis rate was calculated by the ratio of PI to DAPI fluorescence intensity and presented as fold change versus the BSA group. (**C**) Expression of apoptosis-related protein markers measured by Western blot (WB). Data were collected from triplicate independent experiments and were shown as means ± SEM, * *p* < 0.05, ** *p* < 0.01, *** *p* < 0.001 versus BSA control group, # *p* < 0.05, ### *p* < 0.001 versus PA group.

To explore whether C3G could have a protective effect on PA-induced apoptosis, we first checked the cell apoptosis rate by applying both Annexin V-FITC/PI double staining flow cytometry and DAPI/PI fluorescent staining. Compared with the control group, the apoptosis rate was dramatically augmented by 188% under PA exposure, while C3G treatment remarkably reduced the apoptosis rate by 47% (Figure 2A). Further, 50 µM C3G treatment significantly decreased the cell apoptosis by 37% compared with the PA group, as indicated by DAPI/PI fluorescent staining (Figure 2B).

Cell-apoptosis-related proteins, including BAX, BCL2, and cleaved caspase-3, were further detected by WB (Figure 2C). Compared to the PA group, supplementation of C3G downregulated the expression of the pro-apoptotic protein BAX and the apoptotic marker protein cleaved caspase-3 by 21% and 37%, respectively, and upregulated the anti-apoptotic protein BCL2 by 131%. These observations of apoptosis were also consistent with the CCK8 assay (Figure S1B–D).

### 3.2. Exploration of the Possible Molecular Mechanisms in Mediating the Protective Role of C3G

**Figure 3:**
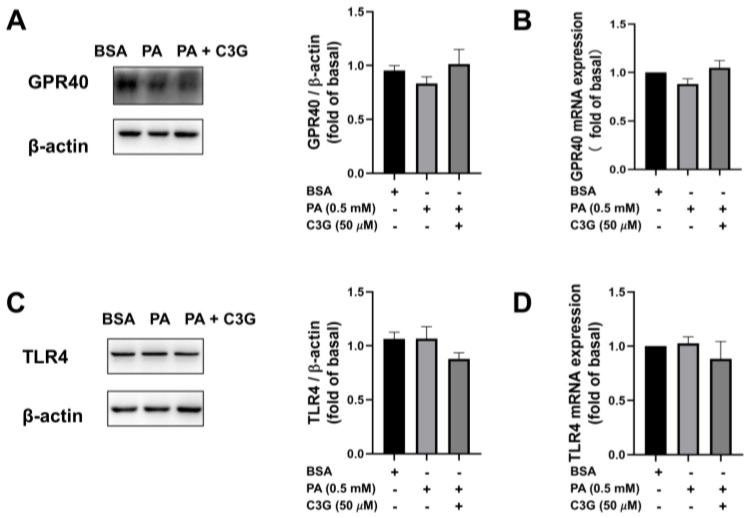
Effects of C3G on mRNA and protein expression of G-protein-coupled receptor 40 (GPR40) and toll-like receptor 4 (TLR4) in treated INS-1E cells. (**A**) WB results of GPR40. (**B**) Quantitative real-time PCR (qPCR) results of GPR40. (**C**) WB results of TLR4. (**D**) The qPCR results of TLR4.

To investigate the molecular mechanisms that mediated the protective effect of C3G on PA-induced beta cell dysfunction, we first used qPCR and WB to detect the expression of cell membrane receptors, including toll-like receptor 4 (TLR4) and G-protein-coupled receptor 40 (GPR40), which recent studies had reported to be involved with beta cell apoptosis and insulin secretory capacity [23,24,25]. No significant change was detected between the PA + C3G group and the PA group, as shown in Figure 3A–D.

**Figure 4:**
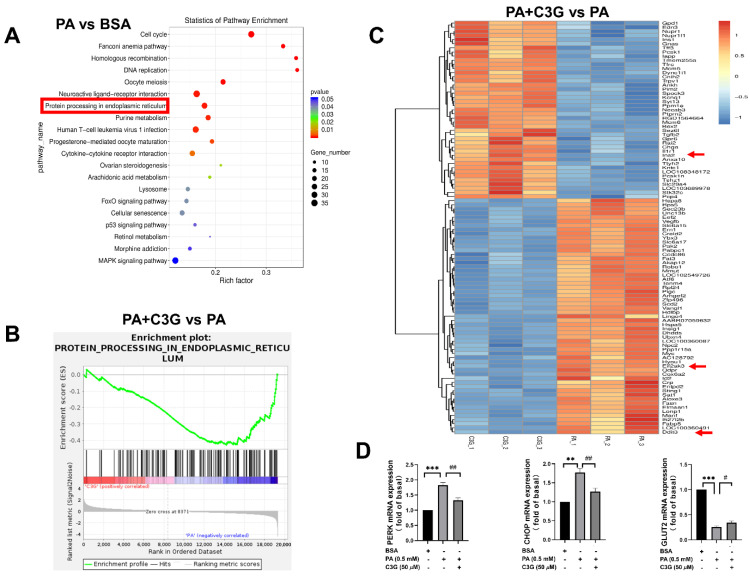
Effects of C3G on differently expressed genes (DEGs) detected by RNA-sequencing (RNA-seq) in treated INS-1E cells. (**A**–**C**) RNA-seq analysis on DEGs among groups (each group with three replicates). (**A**) Kyoto Encyclopedia of Genes and Genomes (KEGG) enrichment plot of PA group versus BSA group. The rich factor indicates the proportion of DEGs in a KEGG pathway. (**B**) Gene Set Enrichment Analysis (GSEA) enrichment plot of the PA + C3G group versus the PA group. (**C**) Heatmap on DEGs of the PA + C3G group versus the PA group. The significantly upregulated genes were in red, while the downregulated ones were in blue. (**D**) The mRNA levels of ER-stress-related genes (PERK and CHOP), and insulin-synthesis-related gene (GLUT2) in INS-1E cells were quantified by qPCR and normalized to the BSA group (*n* = 6). Data were shown as means ± SEM, ** *p* < 0.01, *** *p* < 0.001 versus BSA control group, # *p* < 0.05, ## *p* < 0.01 versus PA group.

Next, RNA-seq was applied to screen the differentially expressed genes, and the results were analyzed using different methods. The pathway enrichment analysis based on the Kyoto Encyclopedia of Genes and Genomes (KEGG) was performed to find the most significantly altered pathways, and GSEA was utilized to quantify the expression of gene sets under relevant pathways to see if the expression was up- or downregulated. As shown in Figure 4A,B, KEGG analysis showed that the endoplasmic reticulum (ER) stress pathway ranked as one of the top pathways in the PA group compared to the control group, while GSEA analysis presented significantly lower expression of gene sets in the ER stress pathway and higher expression in the DNA proliferation and insulin secretion pathway (Figure S2A,B) in the PA + C3G group compared to the PA group, which suggested that the protective effect of C3G on cell apoptosis and insulin secretion was probably accomplished by alleviating ER stress, promoting cell proliferation and insulin secretion pathways. Besides, the volcano plot (Figure S2C) and heatmap (Figure 4C), based on clustering analysis of DEGs, further indicated that the top downregulated ER stress pathway genes involved eif2αk3 (PERK), and ddit3 (CHOP). PERK, CHOP, and GLUT2 expression were further confirmed by qPCR, and that was in line with RNA-seq analysis (Figure 4D). The insulin-secretion-related genes, including ins1, ins2, and slc2a2 (GLUT2), were ranked as the top upregulated genes, consistent with the restored GSIS by C3G treatment.

### 3.3. Verification of the Critical Role of ER Stress Pathway in Mediating the Protective Effect of C3G

**Figure 5:**
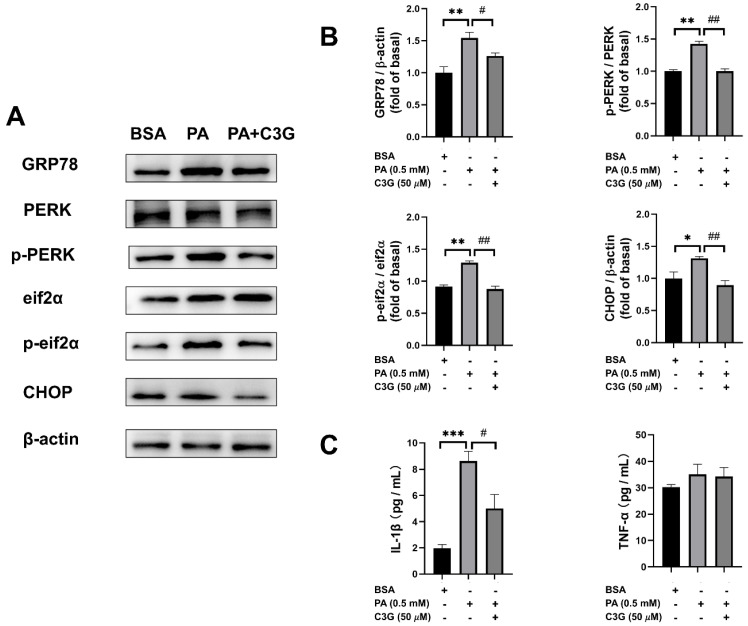
Effects of C3G on protein expression of ER stress signaling pathways and inflammatory factor concentration. (**A**) The ER stress PERK-pathway-related protein levels were measured by WB. (**B**) WB plots were analyzed and the value was normalized to the BSA group. (**C**) Inflammatory factors interleukin-1beta (IL-1β) and tumor necrosis factor-alpha (TNF-α) were measured by ELISA. Experiments were conducted in triplicate and data were shown as means ± SEM, * *p* < 0.05, ** *p* < 0.01, *** *p* < 0.001 versus BSA control group, # *p* < 0.05, ## *p* < 0.01 versus PA group.

The ER stress pathway is mainly mediated by three endoplasmic reticulum transmembrane proteins, including PERK, inositol-requiring enzyme 1 (IRE1), and transcription factor 6 (ATF6) [26,27]. Based on the RNA-seq analysis and qPCR results, we used WB to validate the expression of markers in ER stress pathways. C3G significantly lowered the PA-induced augmentation on the expression of GRP78, CHOP, and the elevated phosphorylation of PERK and eif2α (Figure 5A). Especially for CHOP and phosphorylated eif2α, the inhibitory effect of C3G on the expression of these ER stress markers led to 18–32% reduction (Figure 5B).

We then further tested the secretion of several inflammatory factors and found that interleukin-1beta (IL-1β) secretion was decreased by 42% in the PA + C3G group compared to the PA group (Figure 5C).

### 3.4. Verification of the Critical Role of CHOP-Medicated ER Stress in Mediating the Protective Role of C3G

**Figure 6:**
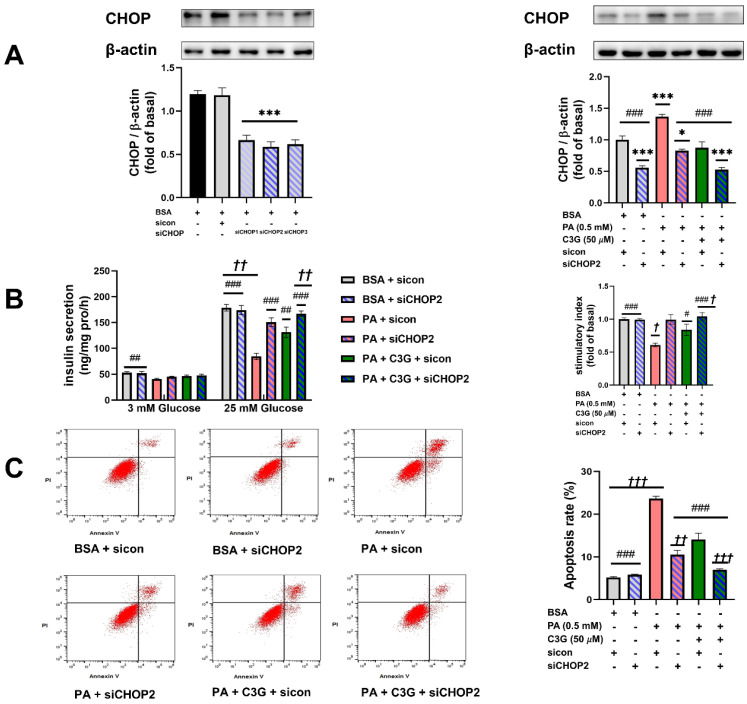
Effects of knockdown of CHOP on PA-induced damage on INS-1E cell GSIS and survival. (**A**) Effects of siCHOP transfection on the CHOP expression in INS-1E cells tested by WB. (**B**) GSIS on INS-1E cells transfected with siCHOP2. (**C**) Cell apoptosis rate of INS-1E cells treated with siCHOP2 tested by flow cytometry. Values were shown as means ± SEM from triplicate experiments, * *p* < 0.05, *** *p* < 0.001 versus BSA + sicon group, # *p* < 0.05, ## *p* < 0.01, ### *p* < 0.001 versus PA + sicon group, *† p* < 0.05, *†† p* < 0.01, *††† p* < 0.001 versus PA + C3G + sicon group.

To verify the critical role of CHOP-medicated ER stress, we applied CHOP siRNA (siCHOP) to suppress the expression of CHOP; scrambled control RNA sicon was applied in the control group. siCHOP2 was selected in subsequent experiments, which induced CHOP reduction by more than 50% (Figure 6A). Compared with the PA + sicon group, knockdown of CHOP could effectively restore the PA-induced reduction in GSIS and increase in apoptosis rate (Figure 6B,C). The PA + C3G + siCHOP2 group exerted a further protective effect on both GSIS and apoptosis rate compared with PA + C3G + sicon group. These findings indicated the center role of CHOP in PA-induced beta cell dysfunction and that played a critical role in mediating the protective effect of C3G.

## 4. Discussion

Anthocyanin C3G has long been interesting as a plant-derived product with anti-diabetic properties, but the molecular mechanism is still not fully understood. In the present study, we demonstrated, for the first time, that C3G could potently restore the pancreatic beta cell’s secretory function and reduce apoptosis under PA exposure. Furthermore, we demonstrated that CHOP-mediated ER stress was the central mechanism of the PA-induced pancreatic beta cell dysfunction and could be effectively restored by C3G supplementation. Conclusively, the study provided a new angle for understanding the anti-diabetic beneficial effect of C3G via preserving pancreatic islets from lipotoxicity.

Previous studies reported that long-term high PA intake caused a significant decrease in serum insulin levels and islet morphology atrophy in wild-type mice and that exposure to a mixture of palmitic and oleic acids triggered an increased rate of apoptosis in INS-1E cells [4,28]. Here, we also observed that long-term exposure to PA directly blunted the glucose-stimulated secretory function in both beta cell line and primary isolated islets and dramatically triggered apoptosis in INS-1E cells. Furthermore, we observed that C3G could dose-dependently improve the impairment of GSIS by PA exposure. The ability of C3G to improve the cell viability or protect beta cells from apoptosis upon various deleterious factors, including high glucose, H_2_O_2_, amylin, or Ab-42, has been observed and reported by several studies [16,29,30,31,32]. In line with these reports, using both flow cytometry and immunostaining, we also observed that supplementation of C3G could significantly reduce the apoptosis induced by PA.

A number of studies have suggested that TLR4 and GPR40, also known as free fatty acid receptor 1 (FFAR1), are expressed on beta cell membranes. It is reported that they could respond to medium- and long-chain saturated fatty acids, and mediate the downstream pathways that play a critical role in FFA-induced beta cell apoptosis and dysfunction [23,24,25,33,34,35]. Therefore, we detected the expression of these two receptors by qPCR and WB. Interestingly, neither of them displayed significant differences following the treatment of C3G, which reminded us to look for other ways to explore the possible mechanism.

It has been reported by recent studies that ER stress has a great impact on pancreatic beta cells, and PERK, one of the three known ER stress transmembrane receptors, is involved with proinsulin folding and cell survival [26,27,36]. The RNA-seq analysis from the present study did indicate that ER stress signaling pathways were the key molecular response to the PA-induced pancreatic beta cell dysfunction and that could be effectively alleviated by C3G supplementation. The other possible pathways are still worth investigating to completely understand the protective role of C3G on pancreatic beta cells in a future study. The activation of PERK and its downstream eif2a, CHOP signaling was further confirmed by WB and C3G supplementation could potently reduce their activation and expression, which was supported by a number of studies, demonstrating a close relationship between increased expression of PERK receptor and downstream eif2α or CHOP and saturated-fatty-acid-induced beta cell apoptosis in rodent and human islets [37,38,39]. Meanwhile, the elevated level of IL-1β further confirmed that ER-stress-related inflammatory pathways were activated by PA exposure, which was consistent with the reported studies describing the inflammatory NLRP3 pathway that interacts with ER stress pathways in diabetes [40,41]. Taken together, these observations indicated that C3G might exert protective effects on the PA-induced beta cell dysfunction through inhibiting the PERK pathway.

Next, we used siRNA to further verify the critical role of CHOP in mediating the protective role of C3G in PA-induced pancreatic cell dysfunction. After knocking down the pro-apoptosis marker of ER stress, both WB and flow cytometry results supported that inhibition of ER stress pathway could suppress cell apoptosis. Interestingly, the combined effect of C3G and siCHOP2 presented the most significant decrease in cell apoptosis, which indicated that C3G did ameliorate beta cell ER stress to cut down the PA-induced damage. The ability of C3G to alleviate ER stress in hepatocytes, endothelial cells, retinal cells, and neuronal cells and, thereby exerting various health-prompting benefits, as reported previously [42,43,44,45]. Here, our data suggested, for the first time, that C3G could play a protective role against ER stress in beta cells and that may preserve the pancreatic beta cell from lipotoxic damage.

Although it is hard to expect the in vitro C3G dosage could be achieved from merely food or dietary supplements [46]. With the rapid progress of nanoencapsulation and the delivery system of food-derived bioactive compounds [47], C3G-based precision nutritional intervention targeting pancreatic beta cell dysfunction in obesity-related diabetes will be of great interest.

## 5. Conclusions

In summary, the data presented in our study demonstrated the protective role of C3G on PA-induced beta cell dysfunction through alleviation of the CHOP-mediated ER stress pathway. Our study provides direct evidence of the protective effect of C3G on pancreatic beta cell dysfunction upon fatty acid stress, which might help to fully understand the anti-diabetic properties of C3G and develop C3G as a nutritional intervention targeting beta cell lipotoxicity in obesity and its related diabetes.

## Data Availability

Not applicable.

## Supplementary Materials

The following supporting information can be downloaded at: https://www.mdpi.com/article/10.3390/nu14091835/s1, Figure S1: Cell viability of INS-1E cells at different Cyanidin-3-O-glucoside (C3G) doses and periods; Figure S2: The Gene Set Enrichment Analysis (GSEA) enrichment plots and the advanced volcano plots from the RNA sequencing analysis.
