# Supplementary material for: Cyanidin-3-O-Glucoside Ameliorates Palmitic-Acid-Induced Pancreatic Beta Cell Dysfunction by Modulating CHOP-Mediated Endoplasmic Reticulum Stress Pathways
Source: Nutrients. 2022 Apr 28;14(9):1835. doi: 10.3390/nu14091835 (PMC9103664; doi:10.3390/nu14091835)
Supplement: Supplementary file 1 [file nutrients-14-01835-s001.zip › nutrients-1671237-supplementary.pdf]

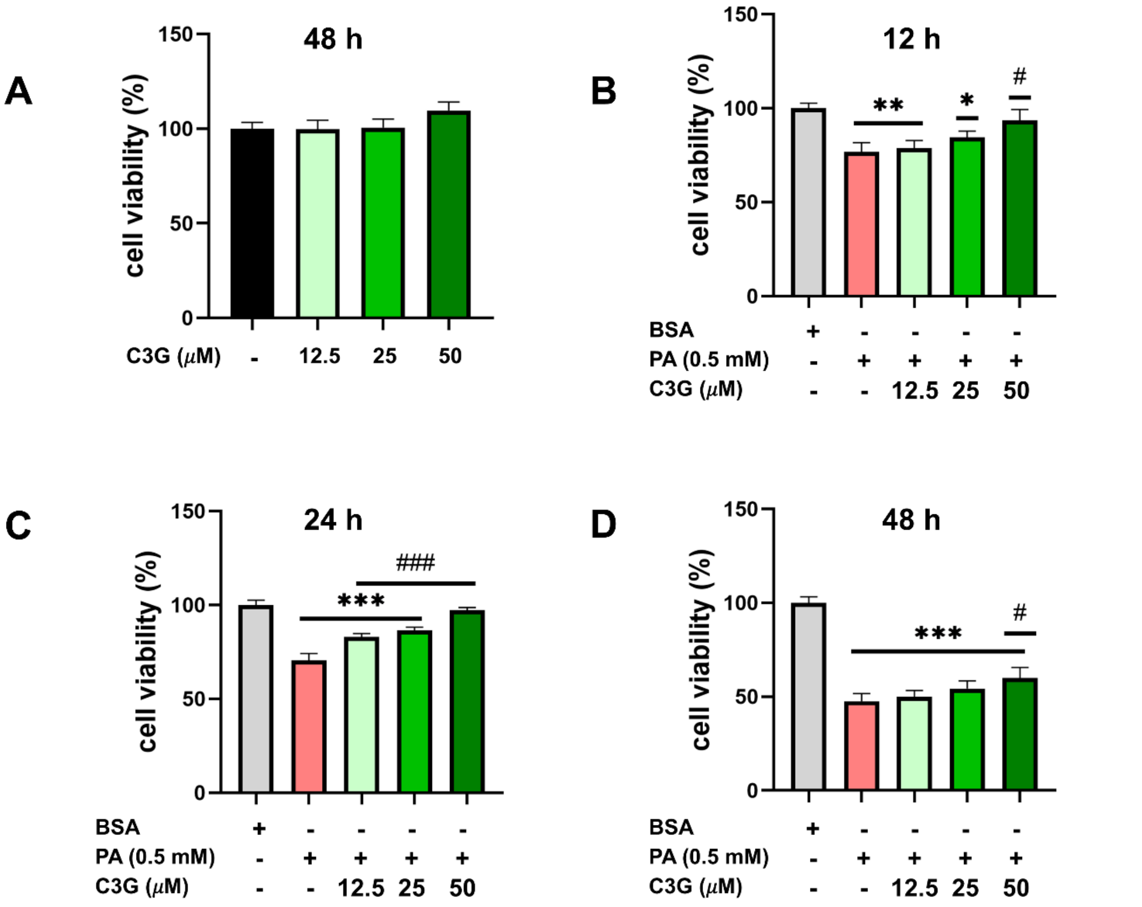

Supplementary Figure S1. Cell viability of INS-1E cells at different Cyanidin-3-O-Glucoside (C3G) doses and periods. (A) Cell viability of INS-1E cells at different doses (12.5 μM, 25 μM, 50 μM) of C3G or only complete cell medium for 48 h measured by CCK8 assay. (B-D) Cell viability on INS-1E cells treated with PA plus 12.5 μM-50 μM C3G measured by CCK8. The results included three periods ranging from 12 h- 48 h. Experiments were performed in triplicate and results were shown as means ± SEM, * $p < 0.05$, ** $p < 0.01$, *** $p < 0.001$ versus BSA control group, # $p < 0.05$, ### $p < 0.001$ versus PA group.

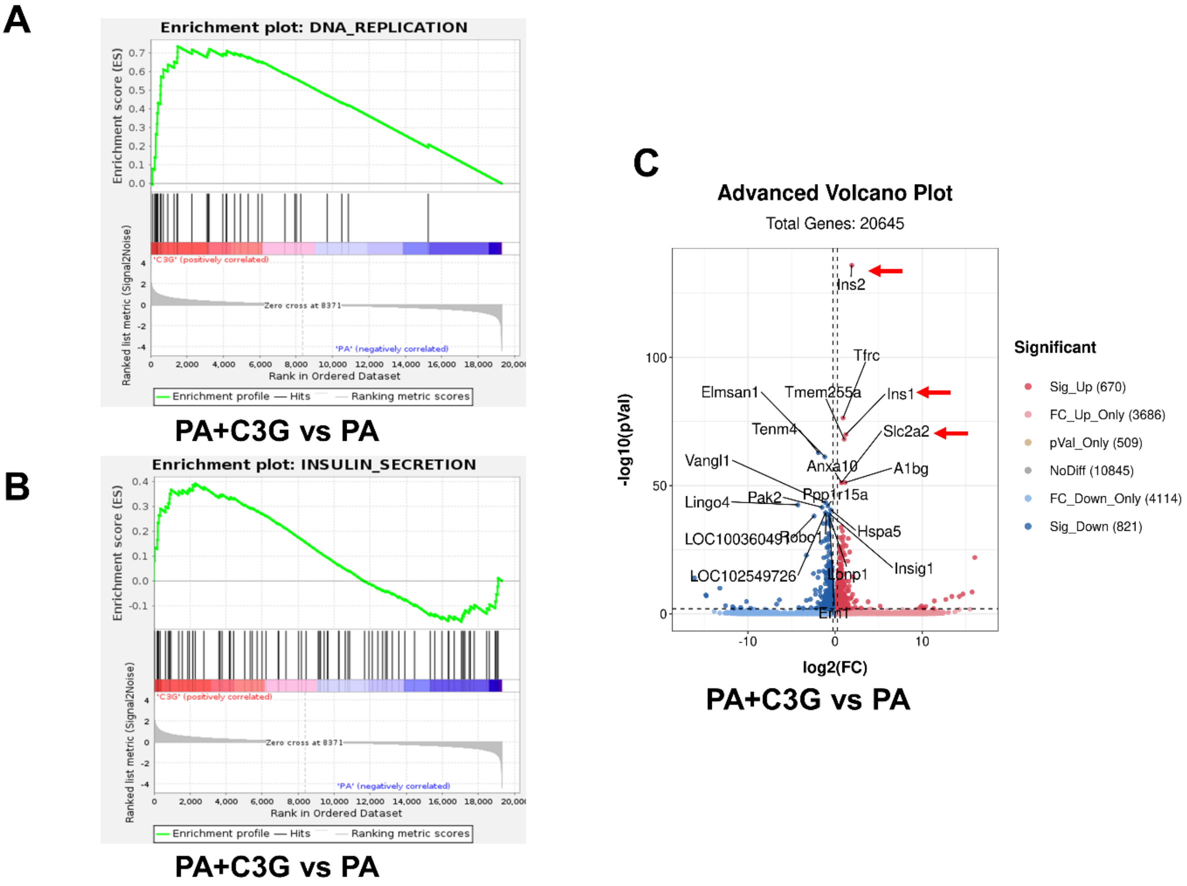

Supplementary Figure S2. The Gene Set Enrichment Analysis (GSEA) enrichment plots and the advanced volcano plots from the RNA sequencing analysis. (A-B) GSEA enrichment plot on DNA proliferation and insulin secretion pathway in PA + C3G group versus the PA group. (C) Volcano plot on differentially expressed genes in PA + C3G group versus the PA group. The significantly up-regulated genes were in red, while the down-regulated ones were in blue.
